# *trans*-Di-μ-acetato-[μ-*N*,*N*-bis­(diphenyl­phosphino)aniline]bis­[chlorido­molybdenum(II)](*Mo—Mo*)–dichloro­methane–tetra­hydro­furan (1/0.3/1.7)

**DOI:** 10.1107/S1600536809007016

**Published:** 2009-03-06

**Authors:** Marko Hapke, Anina Wöhl, Stephan Peitz, Anke Spannenberg, Uwe Rosenthal

**Affiliations:** a Leibniz-Institut für Katalyse e. V. an der Universität Rostock, Albert-Einstein-Strasse 29a, 18059 Rostock, Germany; b Linde AG, Linde Engineering Division, Dr.-Carl-von-Linde-Strasse 6-14, 82049 Pullach, Germany

## Abstract

The mol­ecular structure of the title compound, [Mo_2_(CH_3_COO)_2_Cl_2_(C_30_H_25_NP_2_)]·0.3CH_2_Cl_2_·1.7C_4_H_8_O, features an Mo—Mo dumbbell bridged by two acetate groups which are *trans* to each other. Perpendicular to the plane spanned by the acetate groups, the Ph_2_PN(Ph)PPh_2_ ligand bridges both Mo atoms, having a P—N—P angle of 114.09 (19)°. In a *trans* position to the *PNP* ligand are two Cl atoms, one on each molybdenum centre. The Mo—Mo bond distance is 2.1161 (9) Å, within the range known for Mo—Mo quadruple bonds. The Mo complex is located on a crystallographic twofold rotation axis which runs through the N—C bond of the ligand. The site occupation factors of the disordered solvent molecules were fixed to 0.15 for dichloromethane and 0.85 for tetrahydrofuran.

## Related literature

For derivatives of the title compound, mostly with monodentate phosphane ligands, see Green *et al.* (1982 ▶). For the synthesis and structural evaluation of dimolybdenum species containing two *trans*-standing *PNP* ligands, see: Cotton *et al.* (1996 ▶, 2006 ▶), Arnold *et al.* (1996 ▶), Wu *et al.* (1997 ▶). For the catalytic properties of the PNP ligand systems with middle and late transition metals, see: Wöhl *et al.* (2009 ▶). For the free ligand, see Fei *et al.* (2003 ▶).
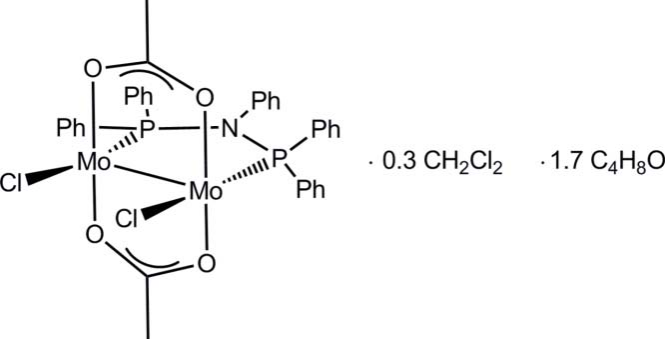


## Experimental

### 

#### Crystal data

[Mo_2_(C_2_H_3_O_2_)_2_Cl_2_(C_30_H_25_NP_2_)]·0.3CH_2_Cl_2_·1.7C_4_H_8_O
*M* *_r_* = 990.37
Monoclinic, 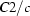

*a* = 15.769 (3) Å
*b* = 13.913 (3) Å
*c* = 20.108 (4) Å
β = 107.32 (3)°
*V* = 4211.3 (15) Å^3^
*Z* = 4
Mo *K*α radiation
μ = 0.88 mm^−1^
*T* = 200 K
0.20 × 0.15 × 0.10 mm

#### Data collection

Stoe IPDS-II diffractometer
Absorption correction: none
33618 measured reflections
4834 independent reflections
3695 reflections with *I* > 2σ(*I*)
*R*_int_ = 0.076

#### Refinement

*R*[*F* ^2^ > 2σ(*F* ^2^)] = 0.033
*wR*(*F* ^2^) = 0.076
*S* = 0.90
4834 reflections
263 parameters
22 restraints
H-atom parameters constrained
Δρ_max_ = 0.99 e Å^−3^
Δρ_min_ = −0.72 e Å^−3^

### 

Data collection: *X-AREA* (Stoe & Cie, 2005 ▶); cell refinement: *X-AREA*; data reduction: *X-RED* (Stoe & Cie, 2005 ▶); program(s) used to solve structure: *SIR2004* (Burla *et al.*, 2005 ▶); program(s) used to refine structure: *SHELXL97* (Sheldrick, 2008 ▶); molecular graphics: *SHELXTL* (Sheldrick, 2008 ▶); software used to prepare material for publication: *SHELXTL*.

## Supplementary Material

Crystal structure: contains datablocks I, global. DOI: 10.1107/S1600536809007016/bt2886sup1.cif

Structure factors: contains datablocks I. DOI: 10.1107/S1600536809007016/bt2886Isup2.hkl

Additional supplementary materials:  crystallographic information; 3D view; checkCIF report

## Figures and Tables

**Fig. 1.**
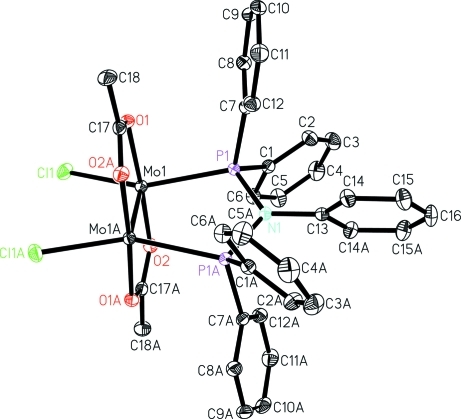
The molecular structure of the title compound showing the atom-labelling scheme (operator for generating equivalent atoms: -x + 2, y, -z + 3/2). Anisotropic displacement ellipsoids are drawn at the 30% probability level. Hydrogen atoms and solvent molecules are omitted for clarity.

## supplementary crystallographic information

### Comment

In the chemistry of molecular compounds containing two metal atoms sharing a metal-metal-bond, chelating ligands have found wide-spread use (Cotton *et al.* 2006). Ligands containing the "PNP" moiety as the structural motif of the coordination unit have been used in a number of cases for the bridging of the metal-metal unit. In all cases, the Mo—Mo bond was symmetrically bridged by two PNP units, which are arranged in *trans*-position. In most cases the common precursor for the preparation of the diphosphine complexes was Mo(OAc)_4_, from which by addition of TMSCl two acetato groups were removed and the free coordination sites occupied by the PNP ligands (Arnold *et al.* 1996, Wu *et al.* 1997 and Cotton *et al.* 1996). We became interested into PNP complexes during our studies on the selective oligomerization of ethene *via* transition metal-catalyzed tri- or tetramerization, yielding 1-hexene or 1-octene (Wöhl *et al.* 2009). Our initial experimental work was focusing on a chromium-based catalyst system (CrCl_3_(THF)_3_/Ph_2_PN(*i*Pr)PPh_2_/MAO) where we also investigated the use of dinuclear chromium complexes. However, for reasons of comparison we wanted to examine comparable molybdenum complexes that contain the PNP ligand moiety. During these experiments we discovered, that by the procedure described below we were able to isolate a molybdenum complex, that contains only one PNP ligand, Ph_2_PN(Ph)PPh_2_, to bridge the two Mo centres, which has to the best of our knowledge not been described yet for PNP ligands. The molecular structure features a Mo—Mo unit which is bridged by two acetato groups which are *trans* to each other (Fig. 1). Perpendicular to the plane spanned by the acetato groups the Ph_2_PN(Ph)PPh_2_ ligand is bridging both Mo atoms, having a P—N—P angle of 114.09 (19)°, nearly the same as found in the free ligand (Fei *et al.* 2003). In *trans*-position to the PNP ligand are two chlorine atoms located, one at each molybdenum center. The Mo—Mo bond distance is 2.1161 (9) Å and within the range known for Mo—Mo quadruple bonds. The asymmetric unit of the title compound contains a half molecule of [(Ph_2_PN(Ph)PPh_2_)(OAC)_2_Cl_2_Mo_2_] besides THF and CH_2_Cl_2_ as lattice solvent with occupancies 0.85:0.15.

### Experimental

Molybdenum(II)-acetate (200 mg, 0.467 mmol) and *N*,*N*-bis(diphenylphosphino)-phenylamine (2.5 equiv., 540 mg, 1.17 mmol) were weighted into a Schlenk flask and 25–30 ml dry THF added. To this green-yellow suspension trimethylsilylchloride (15 equiv., 0.9 ml, 7 mmol) was added *via* syringe and the reaction mixture stirred at room temperature. The colour of the solution was changing to yellow-orange, red and after a couple of minutes she became red-violett. After 10 minutes a red-violett precipitate started to appear while stirring was continued. After standing without stirring over night the reaction mixture was filtrated under argon and the violet solid product washed with 20 ml portions of THF and *n*-hexane twice each. The violet solid was dried in high vacuo giving a fine powder (328 mg). The compound was characterized by NMR (^1^H, ^13^C and ^31^P NMR; solvent: CDCl_3_). Suitable crystals for X-ray analysis have been grown by diffusion of THF into a solution of the complex in CH_2_Cl_2_.

### Refinement

All non-H  atoms excluding the CH_2_Cl_2_ molecule were refined anisotropically. C23, Cl2 and Cl3 were refined isotropically. The site occupation factors of the disordered solvent molecules were fixed to 0.85 for THF and 0.15 for dichloromethane. All H atoms were placed in idealized positions with d(C—H) = 0.99 (CH_2_), 0.98 (CH_3_) and 0.95 Å (CH) and refined using a riding model with *U*_iso_(H) fixed at 1.5 *U*_eq_(C) for CH_3_ and 1.2 *U*_eq_(C) for CH_2_ and CH. Distance restraints (SADI in SHELXL) were used to improve the geometry of THF and CH_2_Cl_2_. Additionally, the anisotropic displacement parameters (SIMU) of C atoms sharing a common bond in the THF molecule were restrained to be equal.

### Crystal data

**Table:**

[Mo_2_(C_2_H_3_O_2_)_2_Cl_2_(C_30_H_25_NP_2_)]·0.3CH_2_Cl_2_·1.7C_4_H_8_O	*F*(000) = 2010
*M**_r_* = 990.37	*D*_x_ = 1.562 Mg m^−^^3^
Monoclinic, *C*2/*c*	Mo *K*α radiation, λ = 0.71073 Å
Hall symbol: -C 2yc	Cell parameters from 20698 reflections
*a* = 15.769 (3) Å	θ = 1.9–29.7°
*b* = 13.913 (3) Å	µ = 0.88 mm^−^^1^
*c* = 20.108 (4) Å	*T* = 200 K
β = 107.32 (3)°	Prism, red
*V* = 4211.3 (15) Å^3^	0.20 × 0.15 × 0.10 mm
*Z* = 4	

### Data collection

**Table:**

Stoe IPDS-II diffractometer	3695 reflections with *I* > 2σ(*I*)
Radiation source: fine-focus sealed tube	*R*_int_ = 0.076
graphite	θ_max_ = 27.5°, θ_min_ = 2.0°
ω scans	*h* = −20→20
33618 measured reflections	*k* = −18→18
4834 independent reflections	*l* = −26→26

### Refinement

**Table:**

Refinement on *F*^2^	Primary atom site location: structure-invariant direct methods
Least-squares matrix: full	Secondary atom site location: difference Fourier map
*R*[*F*^2^ > 2σ(*F*^2^)] = 0.033	Hydrogen site location: inferred from neighbouring sites
*wR*(*F*^2^) = 0.076	H-atom parameters constrained
*S* = 0.90	*w* = 1/[σ^2^(*F*_o_^2^) + (0.0436*P*)^2^] where *P* = (*F*_o_^2^ + 2*F*_c_^2^)/3
4834 reflections	(Δ/σ)_max_ = 0.001
263 parameters	Δρ_max_ = 0.99 e Å^−^^3^
22 restraints	Δρ_min_ = −0.72 e Å^−^^3^

### Special details

**Table:**

Geometry. All e.s.d.'s (except the e.s.d. in the dihedral angle between two l.s. planes) are estimated using the full covariance matrix. The cell e.s.d.'s are taken into account individually in the estimation of e.s.d.'s in distances, angles and torsion angles; correlations between e.s.d.'s in cell parameters are only used when they are defined by crystal symmetry. An approximate (isotropic) treatment of cell e.s.d.'s is used for estimating e.s.d.'s involving l.s. planes.
Refinement. Refinement of *F*^2^ against ALL reflections. The weighted *R*-factor *wR* and goodness of fit *S* are based on *F*^2^, conventional *R*-factors *R* are based on *F*, with *F* set to zero for negative *F*^2^. The threshold expression of *F*^2^ > σ(*F*^2^) is used only for calculating *R*-factors(gt) *etc*. and is not relevant to the choice of reflections for refinement. *R*-factors based on *F*^2^ are statistically about twice as large as those based on *F*, and *R*- factors based on ALL data will be even larger.

### Fractional atomic coordinates and isotropic or equivalent isotropic displacement parameters (Å^2^)

**Table:**

	*x*	*y*	*z*	*U*_iso_*/*U*_eq_	Occ. (<1)
C23	0.3316 (18)	0.371 (2)	−0.0151 (15)	0.038 (6)*	0.15
H23A	0.3312	0.3382	0.0286	0.046*	0.15
H23B	0.3456	0.3216	−0.0458	0.046*	0.15
Cl2	0.4164 (8)	0.4554 (9)	0.0049 (6)	0.094 (3)*	0.15
Cl3	0.1952 (10)	0.4261 (14)	−0.0668 (7)	0.075 (3)*	0.15
C1	0.9245 (2)	0.6337 (2)	0.60553 (15)	0.0224 (6)	
C2	0.8729 (2)	0.7137 (2)	0.57764 (17)	0.0314 (7)	
H2	0.8335	0.7400	0.6005	0.038*	
C3	0.8792 (3)	0.7547 (3)	0.51670 (18)	0.0387 (8)	
H3	0.8430	0.8084	0.4973	0.046*	
C4	0.9374 (3)	0.7184 (3)	0.48376 (18)	0.0402 (8)	
H4	0.9416	0.7475	0.4421	0.048*	
C5	0.9894 (3)	0.6402 (3)	0.51100 (17)	0.0359 (8)	
H5	1.0299	0.6156	0.4884	0.043*	
C6	0.9827 (2)	0.5971 (2)	0.57136 (15)	0.0288 (6)	
H6	1.0179	0.5424	0.5896	0.035*	
C7	0.81890 (18)	0.6061 (2)	0.69921 (15)	0.0222 (5)	
C8	0.7406 (2)	0.5965 (2)	0.64439 (17)	0.0289 (6)	
H8	0.7430	0.5795	0.5992	0.035*	
C9	0.6595 (2)	0.6117 (2)	0.6561 (2)	0.0353 (7)	
H9	0.6063	0.6044	0.6187	0.042*	
C10	0.6545 (2)	0.6371 (2)	0.7204 (2)	0.0366 (8)	
H10	0.5984	0.6492	0.7272	0.044*	
C11	0.7316 (2)	0.6452 (2)	0.7757 (2)	0.0365 (8)	
H11	0.7284	0.6623	0.8206	0.044*	
C12	0.8134 (2)	0.6282 (2)	0.76538 (17)	0.0282 (7)	
H12	0.8660	0.6317	0.8037	0.034*	
C13	1.0000	0.7507 (3)	0.7500	0.0267 (9)	
C14	0.9682 (2)	0.8011 (2)	0.7974 (2)	0.0369 (8)	
H14	0.9462	0.7675	0.8299	0.044*	
C15	0.9689 (3)	0.9006 (3)	0.7968 (3)	0.0540 (10)	
H15	0.9475	0.9349	0.8294	0.065*	
C16	1.0000	0.9505 (4)	0.7500	0.0633 (19)	
H16	1.0000	1.0187	0.7500	0.076*	
C17	0.85381 (19)	0.3927 (2)	0.79476 (16)	0.0246 (6)	
C18	0.7734 (2)	0.3832 (2)	0.81938 (19)	0.0344 (8)	
H18A	0.7293	0.4316	0.7963	0.052*	
H18B	0.7904	0.3928	0.8699	0.052*	
H18C	0.7479	0.3188	0.8080	0.052*	
Cl1	0.93542 (6)	0.23863 (6)	0.65324 (4)	0.03252 (18)	
Mo1	0.958490 (16)	0.400753 (17)	0.697846 (12)	0.01851 (7)	
N1	1.0000	0.6457 (2)	0.7500	0.0215 (7)	
O1	0.84401 (13)	0.38946 (15)	0.72997 (11)	0.0243 (4)	
O2	1.07046 (13)	0.40319 (15)	0.66069 (10)	0.0247 (4)	
P1	0.92644 (5)	0.57906 (5)	0.68785 (4)	0.01836 (15)	
O3	0.2246 (4)	0.4448 (4)	0.0103 (3)	0.0979 (17)	0.85
C19	0.1975 (9)	0.4284 (11)	−0.0537 (6)	0.112 (2)	0.85
H19A	0.1902	0.4890	−0.0807	0.134*	0.85
H19B	0.1401	0.3938	−0.0664	0.134*	0.85
C20	0.2727 (5)	0.3642 (6)	−0.0674 (5)	0.1111 (19)	0.85
H20A	0.2642	0.2953	−0.0590	0.133*	0.85
H20B	0.2768	0.3727	−0.1153	0.133*	0.85
C21	0.3560 (7)	0.4072 (6)	−0.0112 (4)	0.1117 (19)	0.85
H21A	0.3885	0.4538	−0.0317	0.134*	0.85
H21B	0.3971	0.3564	0.0139	0.134*	0.85
C22	0.3065 (6)	0.4577 (6)	0.0368 (5)	0.1097 (19)	0.85
H22A	0.3263	0.4299	0.0842	0.132*	0.85
H22B	0.3200	0.5273	0.0402	0.132*	0.85

### Atomic displacement parameters (Å^2^)

**Table:**

	*U*^11^	*U*^22^	*U*^33^	*U*^12^	*U*^13^	*U*^23^
C1	0.0244 (15)	0.0238 (13)	0.0181 (13)	−0.0015 (11)	0.0048 (11)	0.0007 (11)
C2	0.0309 (17)	0.0338 (17)	0.0303 (16)	0.0057 (13)	0.0105 (14)	0.0055 (13)
C3	0.043 (2)	0.0373 (18)	0.0342 (18)	0.0079 (16)	0.0095 (16)	0.0154 (15)
C4	0.056 (2)	0.0410 (19)	0.0262 (16)	−0.0023 (17)	0.0164 (16)	0.0088 (15)
C5	0.049 (2)	0.0379 (18)	0.0269 (16)	0.0041 (15)	0.0212 (16)	0.0022 (14)
C6	0.0342 (16)	0.0287 (14)	0.0244 (14)	0.0008 (14)	0.0101 (12)	0.0002 (13)
C7	0.0200 (13)	0.0201 (13)	0.0274 (14)	0.0030 (11)	0.0084 (11)	0.0029 (12)
C8	0.0263 (15)	0.0279 (15)	0.0312 (15)	0.0001 (13)	0.0065 (12)	0.0036 (14)
C9	0.0209 (15)	0.0302 (17)	0.051 (2)	−0.0003 (13)	0.0051 (14)	0.0109 (15)
C10	0.0255 (17)	0.0290 (16)	0.062 (2)	0.0052 (13)	0.0224 (17)	0.0091 (16)
C11	0.038 (2)	0.0346 (18)	0.046 (2)	0.0028 (15)	0.0255 (17)	0.0008 (15)
C12	0.0257 (16)	0.0307 (16)	0.0295 (16)	0.0012 (12)	0.0102 (13)	−0.0018 (12)
C13	0.021 (2)	0.022 (2)	0.033 (2)	0.000	−0.0004 (17)	0.000
C14	0.0351 (19)	0.0263 (16)	0.047 (2)	0.0040 (14)	0.0081 (15)	−0.0044 (15)
C15	0.050 (2)	0.0286 (18)	0.082 (3)	0.0070 (18)	0.018 (2)	−0.013 (2)
C16	0.053 (4)	0.018 (2)	0.115 (6)	0.000	0.020 (4)	0.000
C17	0.0256 (15)	0.0190 (13)	0.0329 (15)	0.0001 (12)	0.0142 (12)	−0.0007 (12)
C18	0.0268 (16)	0.0380 (19)	0.0456 (19)	−0.0021 (13)	0.0218 (15)	−0.0013 (15)
Cl1	0.0342 (4)	0.0274 (4)	0.0401 (4)	−0.0066 (3)	0.0173 (3)	−0.0114 (3)
Mo1	0.01940 (12)	0.01871 (11)	0.01876 (11)	0.00004 (10)	0.00775 (8)	−0.00079 (10)
N1	0.0215 (17)	0.0169 (16)	0.0244 (17)	0.000	0.0043 (14)	0.000
O1	0.0209 (10)	0.0244 (10)	0.0297 (11)	−0.0014 (8)	0.0108 (8)	−0.0007 (9)
O2	0.0267 (11)	0.0268 (10)	0.0242 (10)	0.0012 (9)	0.0133 (8)	0.0008 (9)
P1	0.0183 (3)	0.0193 (3)	0.0178 (3)	0.0008 (2)	0.0057 (3)	0.0006 (3)
O3	0.075 (3)	0.140 (5)	0.076 (3)	0.007 (3)	0.018 (3)	0.049 (3)
C19	0.110 (4)	0.079 (4)	0.124 (4)	0.013 (3)	−0.001 (4)	0.020 (4)
C20	0.112 (4)	0.078 (3)	0.119 (4)	0.016 (3)	−0.004 (3)	0.020 (3)
C21	0.115 (4)	0.080 (3)	0.113 (4)	0.019 (3)	−0.005 (3)	0.024 (3)
C22	0.118 (4)	0.080 (3)	0.109 (4)	0.020 (3)	−0.001 (3)	0.025 (3)

### Geometric parameters (Å, °)

**Table:**

C23—Cl2	1.74 (3)	C14—H14	0.9500
C23—Cl3	2.23 (3)	C15—C16	1.372 (5)
C23—H23A	0.9900	C15—H15	0.9500
C23—H23B	0.9900	C16—C15^i^	1.372 (5)
C1—C2	1.395 (4)	C16—H16	0.9500
C1—C6	1.396 (4)	C17—O1	1.266 (4)
C1—P1	1.813 (3)	C17—O2^i^	1.270 (4)
C2—C3	1.382 (5)	C17—C18	1.498 (4)
C2—H2	0.9500	C18—H18A	0.9800
C3—C4	1.378 (5)	C18—H18B	0.9800
C3—H3	0.9500	C18—H18C	0.9800
C4—C5	1.375 (5)	Cl1—Mo1	2.4146 (9)
C4—H4	0.9500	Mo1—O1	2.096 (2)
C5—C6	1.385 (4)	Mo1—O2	2.1124 (19)
C5—H5	0.9500	Mo1—Mo1^i^	2.1161 (9)
C6—H6	0.9500	Mo1—P1	2.5278 (9)
C7—C12	1.393 (4)	N1—P1	1.704 (2)
C7—C8	1.395 (4)	N1—P1^i^	1.704 (2)
C7—P1	1.817 (3)	O2—C17^i^	1.270 (4)
C8—C9	1.384 (4)	O3—C19	1.251 (8)
C8—H8	0.9500	O3—C22	1.255 (8)
C9—C10	1.365 (5)	C19—C20	1.573 (8)
C9—H9	0.9500	C19—H19A	0.9900
C10—C11	1.386 (6)	C19—H19B	0.9900
C10—H10	0.9500	C20—C21	1.573 (8)
C11—C12	1.386 (4)	C20—H20A	0.9900
C11—H11	0.9500	C20—H20B	0.9900
C12—H12	0.9500	C21—C22	1.575 (8)
C13—C14^i^	1.390 (4)	C21—H21A	0.9900
C13—C14	1.390 (4)	C21—H21B	0.9900
C13—N1	1.461 (5)	C22—H22A	0.9900
C14—C15	1.384 (5)	C22—H22B	0.9900
			
Cl2—C23—Cl3	116.3 (16)	O1—C17—C18	118.7 (3)
Cl2—C23—H23A	108.2	O2^i^—C17—C18	119.2 (3)
Cl3—C23—H23A	108.2	C17—C18—H18A	109.5
Cl2—C23—H23B	108.2	C17—C18—H18B	109.5
Cl3—C23—H23B	108.2	H18A—C18—H18B	109.5
H23A—C23—H23B	107.4	C17—C18—H18C	109.5
C2—C1—C6	118.9 (3)	H18A—C18—H18C	109.5
C2—C1—P1	123.4 (2)	H18B—C18—H18C	109.5
C6—C1—P1	117.5 (2)	O1—Mo1—O2	175.75 (8)
C3—C2—C1	120.0 (3)	O1—Mo1—Mo1^i^	91.72 (6)
C3—C2—H2	120.0	O2—Mo1—Mo1^i^	90.85 (6)
C1—C2—H2	120.0	O1—Mo1—Cl1	89.78 (6)
C4—C3—C2	120.6 (3)	O2—Mo1—Cl1	86.16 (6)
C4—C3—H3	119.7	Mo1^i^—Mo1—Cl1	110.39 (2)
C2—C3—H3	119.7	O1—Mo1—P1	85.87 (6)
C5—C4—C3	120.2 (3)	O2—Mo1—P1	97.15 (6)
C5—C4—H4	119.9	Mo1^i^—Mo1—P1	97.405 (18)
C3—C4—H4	119.9	Cl1—Mo1—P1	151.98 (3)
C4—C5—C6	119.9 (3)	C13—N1—P1	122.96 (10)
C4—C5—H5	120.0	C13—N1—P1^i^	122.95 (10)
C6—C5—H5	120.0	P1—N1—P1^i^	114.09 (19)
C5—C6—C1	120.5 (3)	C17—O1—Mo1	117.50 (19)
C5—C6—H6	119.8	C17^i^—O2—Mo1	117.26 (18)
C1—C6—H6	119.8	N1—P1—C1	105.33 (12)
C12—C7—C8	118.9 (3)	N1—P1—C7	104.61 (11)
C12—C7—P1	119.5 (2)	C1—P1—C7	105.38 (14)
C8—C7—P1	121.4 (2)	N1—P1—Mo1	113.53 (10)
C9—C8—C7	119.8 (3)	C1—P1—Mo1	115.62 (10)
C9—C8—H8	120.1	C7—P1—Mo1	111.43 (10)
C7—C8—H8	120.1	C19—O3—C22	117.0 (9)
C10—C9—C8	121.1 (3)	O3—C19—C20	103.9 (8)
C10—C9—H9	119.4	O3—C19—H19A	111.0
C8—C9—H9	119.4	C20—C19—H19A	111.0
C9—C10—C11	119.8 (3)	O3—C19—H19B	111.0
C9—C10—H10	120.1	C20—C19—H19B	111.0
C11—C10—H10	120.1	H19A—C19—H19B	109.0
C12—C11—C10	120.0 (3)	C21—C20—C19	99.7 (8)
C12—C11—H11	120.0	C21—C20—H20A	111.8
C10—C11—H11	120.0	C19—C20—H20A	111.8
C11—C12—C7	120.4 (3)	C21—C20—H20B	111.8
C11—C12—H12	119.8	C19—C20—H20B	111.8
C7—C12—H12	119.8	H20A—C20—H20B	109.5
C14^i^—C13—C14	119.4 (4)	C20—C21—C22	98.6 (7)
C14^i^—C13—N1	120.3 (2)	C20—C21—H21A	112.0
C14—C13—N1	120.3 (2)	C22—C21—H21A	112.0
C15—C14—C13	119.6 (4)	C20—C21—H21B	112.0
C15—C14—H14	120.2	C22—C21—H21B	112.0
C13—C14—H14	120.2	H21A—C21—H21B	109.7
C16—C15—C14	121.1 (4)	O3—C22—C21	108.2 (7)
C16—C15—H15	119.5	O3—C22—H22A	110.1
C14—C15—H15	119.5	C21—C22—H22A	110.1
C15^i^—C16—C15	119.2 (5)	O3—C22—H22B	110.1
C15^i^—C16—H16	120.4	C21—C22—H22B	110.1
C15—C16—H16	120.4	H22A—C22—H22B	108.4
O1—C17—O2^i^	122.1 (3)		
			
C6—C1—C2—C3	0.8 (5)	C13—N1—P1—C7	−64.90 (10)
P1—C1—C2—C3	176.0 (3)	P1^i^—N1—P1—C7	115.09 (10)
C1—C2—C3—C4	−1.3 (6)	C13—N1—P1—Mo1	173.409 (15)
C2—C3—C4—C5	0.6 (6)	P1^i^—N1—P1—Mo1	−6.593 (15)
C3—C4—C5—C6	0.6 (6)	C2—C1—P1—N1	−83.1 (3)
C4—C5—C6—C1	−1.1 (5)	C6—C1—P1—N1	92.2 (3)
C2—C1—C6—C5	0.4 (5)	C2—C1—P1—C7	27.2 (3)
P1—C1—C6—C5	−175.1 (3)	C6—C1—P1—C7	−157.5 (2)
C12—C7—C8—C9	1.9 (5)	C2—C1—P1—Mo1	150.7 (2)
P1—C7—C8—C9	175.9 (2)	C6—C1—P1—Mo1	−34.0 (3)
C7—C8—C9—C10	0.6 (5)	C12—C7—P1—N1	−31.0 (3)
C8—C9—C10—C11	−1.8 (5)	C8—C7—P1—N1	155.1 (2)
C9—C10—C11—C12	0.5 (5)	C12—C7—P1—C1	−141.8 (2)
C10—C11—C12—C7	2.1 (5)	C8—C7—P1—C1	44.3 (3)
C8—C7—C12—C11	−3.3 (5)	C12—C7—P1—Mo1	92.1 (2)
P1—C7—C12—C11	−177.3 (2)	C8—C7—P1—Mo1	−81.8 (3)
C14^i^—C13—C14—C15	−0.2 (3)	O1—Mo1—P1—N1	109.43 (7)
N1—C13—C14—C15	179.8 (3)	O2—Mo1—P1—N1	−73.57 (7)
C13—C14—C15—C16	0.4 (6)	Mo1^i^—Mo1—P1—N1	18.23 (4)
C14—C15—C16—C15^i^	−0.2 (3)	Cl1—Mo1—P1—N1	−168.85 (5)
C14^i^—C13—N1—P1	−74.23 (17)	O1—Mo1—P1—C1	−128.66 (13)
C14—C13—N1—P1	105.77 (17)	O2—Mo1—P1—C1	48.34 (13)
C14^i^—C13—N1—P1^i^	105.77 (17)	Mo1^i^—Mo1—P1—C1	140.14 (11)
C14—C13—N1—P1^i^	−74.23 (17)	Cl1—Mo1—P1—C1	−46.94 (13)
O2^i^—C17—O1—Mo1	0.6 (4)	O1—Mo1—P1—C7	−8.37 (12)
C18—C17—O1—Mo1	−179.0 (2)	O2—Mo1—P1—C7	168.62 (11)
Mo1^i^—Mo1—O1—C17	4.7 (2)	Mo1^i^—Mo1—P1—C7	−99.57 (11)
Cl1—Mo1—O1—C17	115.1 (2)	Cl1—Mo1—P1—C7	73.34 (12)
P1—Mo1—O1—C17	−92.6 (2)	C22—O3—C19—C20	37.3 (15)
Mo1^i^—Mo1—O2—C17^i^	7.9 (2)	O3—C19—C20—C21	−32.7 (13)
Cl1—Mo1—O2—C17^i^	−102.4 (2)	C19—C20—C21—C22	18.7 (10)
P1—Mo1—O2—C17^i^	105.5 (2)	C19—O3—C22—C21	−23.9 (12)
C13—N1—P1—C1	45.93 (11)	C20—C21—C22—O3	−1.3 (9)
P1^i^—N1—P1—C1	−134.07 (11)		

Symmetry codes: (i) −*x*+2, *y*, −*z*+3/2.
